# Sequence Polymorphisms and Structural Variations among Four Grapevine (*Vitis vinifera* L.) Cultivars Representing Sardinian Agriculture

**DOI:** 10.3389/fpls.2017.01279

**Published:** 2017-07-20

**Authors:** Luca Mercenaro, Giovanni Nieddu, Andrea Porceddu, Mario Pezzotti, Salvatore Camiolo

**Affiliations:** ^1^Dipartimento di Agraria, Università degli Studi di Sassari Sassari, Italy; ^2^Dipartimento di Biotecnologie, Università degli Studi di Verona Verona, Italy

**Keywords:** *Vitis vinifera*, next generation sequencing, structural variation, SNP, CNV, Run of homozygosity

## Abstract

The genetic diversity among grapevine (*Vitis vinifera* L.) cultivars that underlies differences in agronomic performance and wine quality reflects the accumulation of single nucleotide polymorphisms (SNPs) and small indels as well as larger genomic variations. A combination of high throughput sequencing and mapping against the grapevine reference genome allows the creation of comprehensive sequence variation maps. We used next generation sequencing and bioinformatics to generate an inventory of SNPs and small indels in four widely cultivated Sardinian grape cultivars (Bovale sardo, Cannonau, Carignano and Vermentino). More than 3,200,000 SNPs were identified with high statistical confidence. Some of the SNPs caused the appearance of premature stop codons and thus identified putative pseudogenes. The analysis of SNP distribution along chromosomes led to the identification of large genomic regions with uninterrupted series of homozygous SNPs. We used a digital comparative genomic hybridization approach to identify 6526 genomic regions with significant differences in copy number among the four cultivars compared to the reference sequence, including 81 regions shared between all four cultivars and 4953 specific to single cultivars (representing 1.2 and 75.9% of total copy number variation, respectively). Reads mapping at a distance that was not compatible with the insert size were used to identify a dataset of putative large deletions with cultivar Cannonau revealing the highest number. The analysis of genes mapping to these regions provided a list of candidates that may explain some of the phenotypic differences among the Bovale sardo, Cannonau, Carignano and Vermentino cultivars.

## Introduction

Grapevine berries (*Vitis* spp.) are marketed worldwide as wine, fresh and dried fruits, and as ingredients for cosmetics and nutraceuticals^1^. These diverse applications are possible due to the broad genetic basis of cultivated grapevine germplasm (Laucou et al., 2011; Emanuelli et al., 2013; Maul et al., 2015), which has been propagated independently by many civilizations throughout history (Imazio et al., 2006; This et al., 2006). There are now thousands of cultivated varieties, many grown in the traditional wine-producing countries of Europe, which have arisen by spontaneous mutation, hybridization, self-fertilization, and interactions with viruses (Arroyo-Garcìa et al., 2006). There is significant evidence of introgression from wild vine (*Vitis vinifera europaea* subsp. *sylvestris*) in current commercial cultivars (Sefc et al., 2003; Crespan, 2004; Myles et al., 2011).

The domesticated grapevine is thought to have originated in the Mediterranean (Zohary, 1995; Imazio et al., 2006) although a secondary center of domestication has been proposed in the western Mediterranean (Grassi et al., 2003; Chessa and Nieddu, 2005; Arroyo-Garcìa et al., 2006). The high diversity of local germplasm reflects the domestication of the wild relatives and has been conditioned by vegetative propagation and the repeated introduction of cuttings and plants (Myles et al., 2011). One of the proposed secondary origins is the island of Sardinia, the second largest island in the Mediterranean Sea (Grassi et al., 2003; Chessa and Nieddu, 2005). This ancient civilization was among the first in the western part of the basin to cultivate grapevine and process the berries (Ucchesu et al., 2015, 2016). Wild vines still grow near hundreds of Sardinian rivers, wetlands and commercial vineyards, and the berries are often used to make wine. The extant Sardinian grapevine germplasm includes hundreds of cultivars differing in agronomic performance, berry chemical composition and oenological potential (Castia et al., 1992; Calb et al., 2006; Vacca et al., 2009; Nieddu, 2011). Even so, only 26 traditional cultivars are recorded in the national grape varietal register, although dozens more can be found in older Sardinian vineyards. Despite the large number of cultivars present in Sardinia, few are routinely used for wine production. The amount of land dedicated to viticulture in Sardinia is 26,000 ha, 85% of which is represented by six cultivars. These include the three major red berry varieties Cannonau, Carignano and Bovale sardo, and the major white berry variety Vermentino (Nieddu, 2011).

Cannonau is the most important red berry cultivar grown in Sardinia (8000 hectares, 29% of the total) and is considered synonymous with Garnacha tinta cultivated in Spain and Grenache noir cultivated in France (di Rovasenda, 1877; Molon, 1906; Viala and Vermorel, 1991). This relationship has been confirmed by ampelographic analysis and the comparison of isoenzymes and molecular markers (Calb et al., 2006; De Mattia et al., 2007; Cipriani et al., 2010). The Grenache family is cultivated on 185,000 ha of land worldwide (Anderson and Aryal, 2013) and the existence of differentiated locally adapted genotypes has been proposed (Ortiz et al., 2004; Mercenaro et al., 2016b), with the sequence diversity of numerous accessions sampled in France, Spain and Italy clustering according to the sampling area (Meneghetti et al., 2011). A comparison between cultivars form Armenia and Georgia (the first areas of grapevine domestication) with European cultivars showed that Cannonau was more closely related to the transcaucasian varieties than to other Italian cultivars (Crespan, 2010). Carignano is grown mostly ungrafted on 2,000 ha of land in south-western Sardinia characterized by extremely sandy soil (Mercenaro et al., 2014). It is genetically similar to the Spanish varieties Carinena and Mazuelo, and the French variety Carignan noir (Mercenaro et al., 2014). These synonymous varieties are cultivated on 80,000 ha of land worldwide (Anderson and Aryal, 2013). Bovale sardo is cultivated on ∼800 ha in central Sardinia and is characterized by extensive intra-varietal differences (Nieddu, 2011). It probably has a local origin (Grassi et al., 2003). Finally, Vermentino is one of the most promising white wine cultivars (ranking fifth by volume of wine sold in Italy). It is traditionally cultivated in the west Mediterranean region and was recently introduced into Australia, South Africa and Argentina. Sardinia and France each cultivate Vermentino on ∼4,200 ha, with further vineyards in Ligury and West Tuscany. Sardinian Vermentino has been awarded DOCG status (Controlled and Guaranteed Denomination of Origin).

The combination of high throughput sequencing technologies and the grapevine reference genome (Jaillon et al., 2007) has facilitated comprehensive sequence analysis in diverse grapevine germplasms. Cultivars with different agronomic and oenological characteristics have been re-sequenced to identify genetic differences underlying the distinct phenotypes (Da Silva et al., 2014; Di Genova et al., 2014; Cardone et al., 2016) and comprehensive sequence variation maps are under construction to link these differences with transcriptomic and metabolomic data, as well as information about grapevine breeding practices (Ray and Satya, 2014). In this regard, availability of NGS data boosted, particularly in the last decade, the identification of candidate genes involved in response to stress (Xu et al., 2014), in the production of essential secondary metabolites (Kim and Buell, 2015) in both model and non-model plants (Unamba et al., 2015). Indeed, Giannuzzi et al. (2011) identified duplications in grapevine cultivar Pinot Noir hosting genes responsible for adaptation and response to environmental changes. The analysis of genomic features in different *V. vinifera* cultivars will expand our knowledge of the evolution of the grapevine genome and will facilitate breeding programs.

Here we report a thorough characterization of genomic sequence variation in four Sardinian cultivars compared to the PN40024 reference genome to determine the genomic characteristics underlying the phenotypic differences among these varieties. SNPs and indels for the four Sardinian cultivars were compared to data from three additional cultivars (Gewurztraminer, Sultanina and Tannat) that are not typical of this island agriculture. The present study aims to characterize the reported cultivars in terms of SNPs/indels, complex structural variations and degree of homozigosity, in order to speculate those features possibly underlying their phenotypic peculiarities.

## Materials and Methods

### Reference Sequence and Annotation

The grapevine reference genome with corresponding annotations and associated gene ontology terms (*V. vinifera*, cv. Pinot noir, PN40024 12× assembly V1 prediction) was downloaded from the Grapevine Genome CRIBI Biotech Center website^2^. We used gff2sequence (Camiolo and Porceddu, 2013) to generate coding sequences (CDSs) and the 5′ and 3′ untranslated regions (UTRs).

### DNA Resequencing

High molecular weight DNA was extracted from nuclei starting form 3–5 g of young leaves of *V. vinifera* cv. Cannonau, Bovale, Carignano and Vermentino, using the procedure described in Zhang et al. (1995) without embedding the nuclei in agarose plugs, but directly performing the lysis of nuclear walls with detergent and proteinase K. Resequencing with an Illumina HiSeq 2000 instrument at the Istituto di Genomica Applicata (IGA, Udine, Italy) produced paired-end short reads of variable length and number (Supplementary Table S1). The produced reads have been deposited in the SRA database with the accession numbers SRR5803837, SRR5803836, SRR5803839 and SRR5803838 for Bovale, Cannonau, Carignano and Vermentino, respectively. Sequence read datasets were quality filtered using the NGS QC toolkit (Patel and Jain, 2012) with default parameters prior to downstream analysis. Quality filtering together with the entire downstream analysis pipeline described below was also carried out on three additional grapevine varieties (Gewurztraminer, Tannat and Sultanina) for comparison. The resequencing data corresponding to these latter three cultivars were retrieved from the Sequence Read Archive (Gewurztraminer, ERR514999; Sultanina, SRR924200; and Tannat, SRR863595 and SRR863618).

### Alignment of Reads and Single Nucleotide Variation Detection

Filtered reads were aligned to the reference genome using Altools (Camiolo et al., 2016) (edit distance = 5% of the read length, base quality cutoff = 10). The embedded Altools Pileup Analyzer module was used to create a pileup formatted file reporting only essential data such as coverage and presence/absence of SNPs/indels at each genomic position. Base information was retrieved only when the corresponding position was covered by at least three reads, whereas SNPs and indels were considered only at positions featuring at least the average coverage and with the polymorphism supported by at least half of this value. The statistical significance of the called SNPs and indels was estimated using VarScan (Koboldt et al., 2012) with default parameters and applying a *p*-value cutoff of 0.05. In order to avoid possible bias in the detection of polymorphisms we only retained unambiguously mapped reads at this stage. Finally a threshold of 0.2 in the allele frequency was applied to minimize the detection of somatic mutation. In this condition only 0.1% of the called polymorphisms proved to feature an excessive depth of coverage (e.g., 6 times the coverage standard deviation) possibly underlining alignment artifacts.

### Genome-Wide Data Visualization

The Altools Sliding Analysis module was used to visualize the alignment statistics along the genome (e.g., coverage, SNPs and indels). Briefly, each chromosome was split into a series of adjacent windows (windows size and step = 20,000 bp) that were investigated in terms of average coverage and polymorphism density. Only positions that were covered by reads were used at this stage. The resulting data were used to plot a genome circular representation using Circos (Krzywinski et al., 2009), with all the reported values normalized to the genome average.

### Depth of Coverage Analysis

The Altools Coverage Analyzer tool was used to detect copy number variation (CNV) and presence/absence variation (PAV) in the grapevine cultivar genomes. False CNV due to known genomic repeats was avoided by comparison with the reference genome. Therefore, we first generated a simulated Illumina paired-end reads dataset for the PN40024 reference genome (average coverage 40×) using DWGSIM^3^ and repeated the alignment and pileup procedures. Simulated reads were used in place of the real resequencing data to reduce the effect of non-homogeneous coverage and hemizygous loci in the reference genome (although we are aware that the usage of real reference genome resequencing data may take into account possible technical artifacts in the reads generation step). We then computed the coverage ratio between the target (all paired reads properly mapping were considered at this stage) and reference genomes in 500-bp adjacent windows. Significant fluctuations in the ratio identified using the DNAcopy algorithm (Seshan and Olshen, 2010) were deemed to explain the CNV. PAV was called if coverage was detected in the reference genome but not in the target. Only structural variations longer than 1000 bp were retained for downstream analyses. We decided not to use the paired end information to detect smaller structural variations, since the used DNA library featured a short insert. Hereafter we define *gains* as those genomic regions featuring a significant higher copy number in the target genome compared to the reference. Similarly we define *losses* as those regions featuring a lower copy number in the target genome (including no copies for zero-coverage areas). It is important to note that such definitions are not intended as statements of phylogenesis because the absence of an outgroup makes it impossible to establish which genome has lost or gained DNA during evolution. For the same reason, no effort was made to infer the copy number of these regions. The Altools Genic Extractor tool was used retrieve the annotated genes within the CNV and PAV regions.

### Large Deletions Analysis

Reads mapping onto the reference produced a sam formatted alignment file (Li et al., 2009) that was used to investigate the occurrence of large deletions. We first filtered the initial dataset by removing all reads mapping at multiple positions (only alignments featuring the XT:A:U tag were retained). This step was necessary to exclude the interpretation of genome duplication events within the same chromosome as large deletions. Paired-end reads mapping at a distance between 10,000 and 1,000,000 bp, e.g., incompatible with the estimated insert size, were then considered to reflect a large deletion event. Only structural variations confirmed by at least three paired-end read mates were used for downstream analysis. Such a task was performed by using the Large deletion finder software within the Altools suite. The Genic Extractor tool was used to retrieve the annotated genes within the large deletions. Large deletions together with CNV were not analyzed for the three outgroup cultivars for the sake of clarity and to keep the focus on the varieties typical of the Sardinian agriculture (although they will be considered for future studies).

### Genic Polymorphism Analysis

Single nucleotide polymorphisms and indels were mapped to the genic portion of each genome using the Altools module Polymorphism Analyzer. This estimated the number of events with the potential to modify polypeptide structures, e.g., non-synonymous substitutions responsible for amino acid replacements or premature stop codons, or indels creating a frameshift in the CDS. Transcripts featuring more than five SNPs were aligned to the corresponding reference gene and *d*n/*d*s was calculated by using scripts incorporating the Biopython (Cock et al., 2009) library cal_dn_ds and using the Maximum Likelihood estimation method. Significance of the selection signal was tested by a Fisher’s exact test.

### Regions Characterized by Extensive Homozygosity

Regions of homozygosity (ROH) were identified using plink (Purcell et al., 2007) with a sliding window of 500 SNPs and a minimal ROH size of 50 kb with one heterozygous or missing SNP allowed for each window. Because ROHs may arise due to hemizygosity, we excluded all ROHs that overlapped regions identified as losses.

### Gene Ontology Studies

The R package topGO was used to carry out single-gene enrichment analysis and to determine ontology codes for biological processes and molecular functions.

## Results

### Alignment Statistics

Paired-end genomic reads representing cultivars Bovale, Cannonau, Carignano and Vermentino were mapped to the PN40024 reference genome and reads representing cultivars Gewurztraminer, Sultanina and Tannat were downloaded from public databases for comparison. As shown in **Table 1**, there was significant diversity among the cultivars in terms of several sequence diversity parameters. The SNP density (number of SNPs per Mbp of covered reference genome sequence) varied from a minimum of 5508.0 for Bovale to a maximum of 8522.1 for Vermentino. The indel density (number of indels per Mbp of covered reference genome sequence) ranged from 213.4 for Gewurztraminer to 728.4 for Vermentino. The ratio of the total number of heterozygous/homozygous SNPs varied from 0.6 for Bovale to more than 2 for Cannonau, Gewurztraminer and Tannat. On the other hand, the ratio of the total number of homozygous/heterozygous indels was ∼2 in most cultivars, although Bovale was exceptional with a ratio of 0.7. Among the varieties cultivated in Sardinia, the highest sequence diversity compared to the reference genome was observed for Vermentino, as confirmed by the lowest number of aligned sites. It is important to note that both the total number of reference bases covered by reads and the total number of reads (e.g., the depth of re-sequencing) differed widely for each cultivar. However, it is unlikely that these factors influenced our diversity estimations significantly because we used a conservative approach in which variant calling was restricted to genomic regions covered by a number of reads at least equal to the average genome coverage. Mild tendencies toward a compositional shift emerged from compositional analysis of the polymorphic sites: the average GC content of the polymorphic sites was lower in the reference genome than in the resequenced cultivars, particularly in the case of Cannonau, Gewurztraminer and Tannat (**Table 1**).

**Table 1:** Polymorphisms statistics for 7 grapevine cultivars (four grown in Sardinia + 3 outgroups).

Statistic	Bovale	Cannonau	Carignano	Vermentino	Gewurztraminer	Sultanina	Tannat
Mapped	133507864	171128054	127619642	96553815	133492555	44984526	173818357
HomoSnps	466108	429341	372596	305746	250232	91629	376951
HeteroSnps	269252	934946	582079	517098	596302	178650	915431
Hetero/Homo SNPs	0.6	2.2	1.6	1.7	2.4	1.9	2.4
%SNPs(^∗^1000000)	5508.0	7972.3	7480.6	8522.1	6341.4	6008.3	7435.2
%HomoSNPs (^∗^1000000)	3491.2	2508.9	2919.6	3166.6	1874.5	2036.9	2168.6
%HeteroSNPs (^∗^1000000)	2016.8	5463.4	4561.0	5355.5	4466.9	3971.4	5266.6
HomoIndel	24246	14879	20644	22473	8590	7536	17924
HeteroIndel	16768	32866	37885	47852	19903	15171	36920
Hetero/Homo INDELs	0.7	2.2	1.8	2.1	2.3	2.0	2.1
%INDELs(^∗^1000000)	307.2	279.0	458.6	728.4	213.4	504.8	315.5
%HomoINDELs (^∗^1000000)	181.6	86.9	161.8	232.8	64.3	167.5	103.1
%HeteroINDELs (^∗^1000000)	125.6	192.1	296.9	495.6	149.1	337.2	212.4
Delta-GC at genome SNP position	3.70	5.16	0.59	0.80	5.27	2.21	4.85

To gain insight into the level of sequence diversity at regions presumably subjected to purifying selection, we extrapolated the sequence polymorphisms within the transcripts (CDS and UTRs). As expected, the polymorphism density was much lower in these regions, particularly in the CDS, where sequences are under greater selective pressure due to their role in protein synthesis (**Table 2**). The density of indels in the CDS was even lower, presumably due to their ability to cause disruptive frameshifts (**Table 2**). In some cultivars, the sequence variation in genic regions was dissimilar to the variation observed at the whole-genome level. Cannonau showed the least genomic variation but the highest SNP density in transcripts, although this trend was not uniform throughout the transcript. Indeed, Cannonau UTRs (but not CDSs) proved to be more polymorphic than the other cultivars with the exception of Gewurztraminer (**Table 2**). Bovale, the most similar to the Pinot noir reference genome at the genic level, ranked second in terms of CDS diversity. Indel density in the CDS was uniform in all cultivars with the exception of the two white berry varieties Vermentino and Sultanina, which showed a remarkably higher number of such polymorphisms in CDSs.

**Table 2:** Polymorphisms statistics for 7 grapevine cultivars (four grown in Sardinia + 3 outgroups).

Statistics	Bovale	Cannonau	Carignano	Vermentino	Gewurztraminer	Sultanina	Tannat
**%SNPs(a)**							
*5UTR:*	2033.5	2283.7	1952.6	2025.8	2474.1	1634.5	1852.0
*3UTR:*	2049.9	2343.2	2104.7	1869.2	2155.9	1528.7	2267.1
*CDS:*	540.9	533.3	525.8	551.5	548.4	535.6	533.7
**%INDELs(a)**							
*5UTR:*	166.3	142.0	148.1	200.3	173.7	162.6	110.2
*3UTR:*	184.3	217.6	198.9	211.8	193.7	171.0	207.2
*CDS:*	5.1	5.2	5.1	8.1	5.4	10.2	5.3
**Hetero/Homo SNPs(^∗^100)**							
*5UTR:*	41.9	258.7	119.1	137.7	248.5	170.2	300.7
*3UTR:*	49.2	248.1	123.6	135.0	256.5	195.4	293.7
*CDS:*	53.4	261.8	137.0	171.1	285.2	409.9	347.5
Homo premature stop codons	98	134	85	44	100	19	77
Total premature stop codons	218	906	353	243	643	204	690
Homo new stop codon	58	90	47	28	56	18	55
Total new stop codon	108	268	114	64	191	41	203

*(a) SNPs and Indels percentage are calculated by dividing the number of polymorphism occurrences by the total length of the polymorphic regions.*

Many SNPs caused the loss or gain of stop codons (**Table 2**). Again, Cannonau was distinguished from the other cultivars with the highest number of both premature and new stop codons in both homozygous and heterozygous genomic regions. In contrast, the two white berry varieties Vermentino and Sultanina showed the lowest number of premature stop codons in both homozygous and heterozygous genomic regions. Most of these genes can be considered as pseudogenes because plant transcripts with premature stop codons are usually targeted for degradation via the nonsense mediated decay pathway. We found 1296 putative pseudogenes among the four Sardinian cultivars, 118 of which contained two or more premature stop codons in at least one cultivar. Among these pseudogenes, 75.6% were specific for one cultivar and only 1.3% were shared by all cultivars.

### Homozygosity Islands

We next investigated the allelic variability of SNPs along chromosomes, seeking ROHs (chromosome regions featuring uninterrupted runs of consecutive homozygous SNPs) which are common features of many resequenced genomes (Ku et al., 2011; Metzger et al., 2015). We set a minimal ROH size of 50 kb with a sliding window of 500 homozygous SNPs, allowing for one missing or heterozygous SNP per window.

**FIGURE 1:**
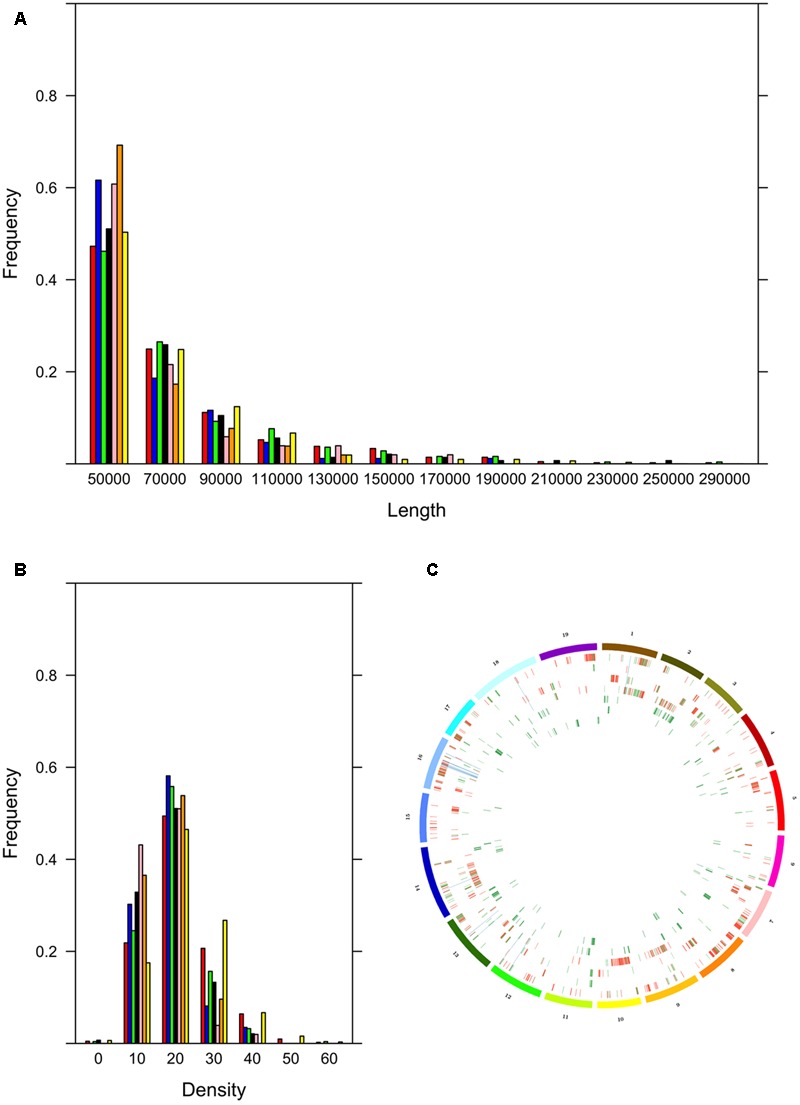
Run of homozygosity (ROH) statistics. **(A)** Frequency distribution based on ROH length in base pairs. **(B)** Frequency distribution based on SNP density within ROH (color codes for sections **A,B**; red: Bovale; blue: Cannonau; green: Carignano; black: Gewurztraminer; pink: Sultanina; orange: Tannat; yellow: Vermentino). **(C)** Distribution of ROHs along the 19 grapevine chromosomes; from outward to inward: Bovale, Cannoanu, Carignano, Vermentino, Gewurztraminer, Sultanina, Tannat; color codes identify ROH regions that are private (red), not private (green) and common to the Sardinian cultivars (blue).

The cultivars could be assigned to two groups, the first with many ROHs (Bovale, Vermentino and Carignano) and the other with few ROHs (Cannonau Sultanina and Tannat) with Gewurztraminer showing intermediate behavior (**Table 3**). As expected, the proportion of the genome included in ROHs was associated with the ROH number. A large proportion of the genome was found within ROHs in the first group: 17.3% in Bovale (34,847,149 bp), 8.6% in Vermentino (24,538,350 bp) and 5.9% in Carignano (18,274,355 bp). A much smaller proportion was found in the second group: 1.2% in Cannonau (4,674,100 bp), 0.9% in Sultanina (3,816,666) and 0.8% in Tannat (3,593,251 bp). The intermediate cultivar Gewurztraminer showed an intermediate proportion of 3.2% (11,412,631 bp). However, there were only minor differences between the two groups in the frequency of ROH distribution. The cultivars with many ROHs tended also to have larger ROHs, whereas those with fewer ROHs tended to have smaller ROHs (**Figure 1A**). Interestingly the frequency distribution of SNP density within ROHs distinguished the two groups more clearly: Carignano, Bovale and Vermentino contained more ROHs with densely clustered SNPs, whereas Cannoanu, Sultanina and Tannat contained more ROHs with sparse SNPs (**Figure 1B**). The ROHs were distributed along all 19 chromosomes, although in a non-uniform manner (**Figure 1C**). Only 16,402 bp of the ROH sequence was common to all cultivars, and this contained 31 protein-coding genes (Supplementary Table S2). An average of 62.5% ROH sequence in each cultivar was private, i.e., restricted to that variety.

**Table 3:** Regions of homozygosity (ROH) statistics for the 7 analyzed grapevine cultivars.

	Total				Private			
Cultivar	#Roh	Total base	Average length	Average SNP density (^∗^100)	#Roh	Total base	Average length	Average SNP density (^∗^100)
Bovale	413	34847149	84500.5	2.7	294	23631639	80379.7	2.8
Cannonau	66	4674100	73332.9	2.4	52	3616265	69543.6	2.5
Carignano	223	18274355	83955.5	2.5	148	12170662	82234.2	2.5
Vermentino	308	24538350	79517.2	2.7	190	14812462	77960.3	2.8
Gewürztraminer	143	11412631	79808.6	2.3				
Sultanina	51	3816666	74836.6	2.1				
Tannat	52	3593251	69101	2.3				

*For the four varieties cultivated in Sardinia, private ROHs are calculated within this group.*

### Structural Variation

**FIGURE 2:**
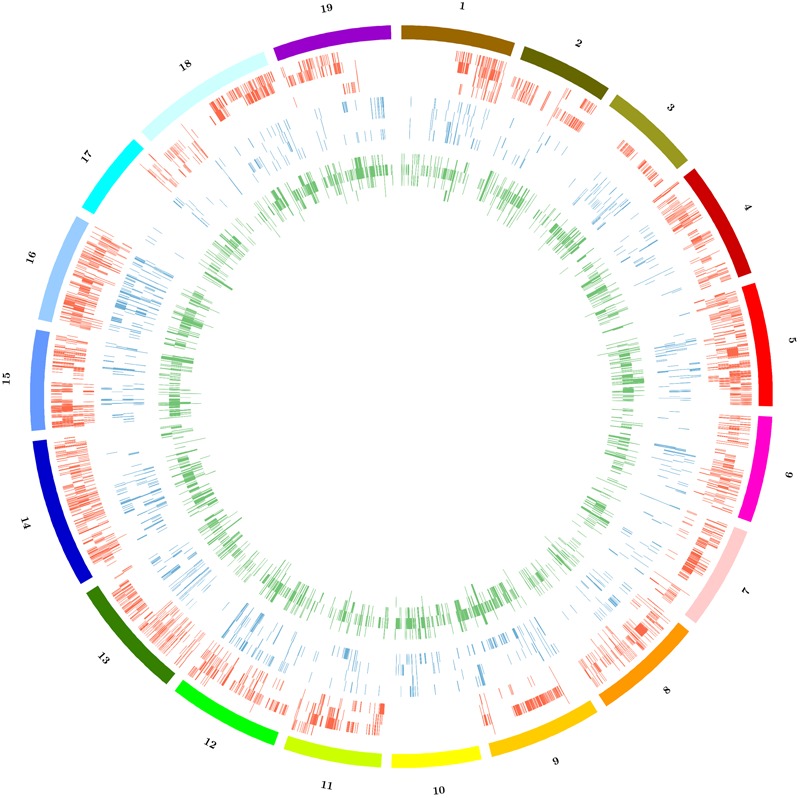
Distribution of copy number variations (CNVs) among the 19 chromosomes of Sardinian cultivars. Color code: red = gains, blue = losses, green = large deletions. For each represented structural variation, the plot represents from outside to inside the cultivars Bovale, Cannonau, Carignano and Vermentino.

Copy number variation, PAV and large deletions are complex structural variations that can be inferred by the analysis of coverage variation along chromosomes. The Altools module Sliding Analysis was used to visualize these variations, and as reported for other resequenced cultivars (Da Silva et al., 2014; Di Genova et al., 2014; Cardone et al., 2016) we found that the coverage was not homogeneous along chromosomes (**Supplementary Figure S1**). We therefore used a digital comparative genome hybridization approach to identify duplicated/deleted genomic regions in the Sardinian cultivars. These were identified as regions with a copy number significantly higher (*gains*) or lower (*losses*) than the corresponding regions in the reference genome. However, we did not determine the actual copy number of these regions in the reference genome so the terms gain and loss are not intended to indicate the direction of mutational events during evolution. We identified 6526 genomic regions with significant differences in copy number among the four cultivars compared to the reference sequence with 81 regions being shared between all four cultivars and 4953 specific to single cultivars. On average, we found that 4.3% of the reference genome was duplicated and 1.4% was deleted in the Sardinian cultivars. Furthermore, 81 of the CNVs (49 gains and 32 losses) corresponding to 316,000 bp (131,000 bp in gains and 185,000 bp in losses) were common to all Sardinian cultivars, whereas 619.1 CNVs were unique to individual cultivars (**Table 4**). The common CNVs encompassed 12 protein-coding genes (Supplementary Table S2). The Cannonau genome contained ninefold more duplicated regions than the Vermentino genome and ∼2.5-fold more than the Carignano and Bovale genomes. In contrast, the Bovale genome showed the highest number of low-copy-number regions followed by Vermentino, Carignano and finally Cannonau (**Table 4**). The length distribution of gains and losses in the Sardinian cultivars is shown in **Supplementary Figure S2**, and **Figure 2** presents a circular genomic map of the distribution of gains and losses along each chromosome. Most of chromosomes 1 and 17 together with the whole of chromosome 10 did not show any gains in any of the cultivars. Vermentino showed the lowest number of chromosomes involved in gain events with chromosomes 14, 3 and 4 featuring only a few such variations. Several common patterns also emerged from the distribution of losses. For example, we observed a common high density of loss events in chromosome 16, but a very low number in chromosome 17. The absence of gains/losses within extended genomic portions must be considered in the light of the DNAcopy algorithm high stringency (e.g., segmentation default *p*-value < 0.01), which can result in a poorer sensitivity. In this regards, applying a higher *p*-value during the genome segmentation step actually resulted in the emergence of previously undetected CNVs events (results not shown). Notably, this phenomenon only apparently affects more the detection of gain compared to loss events (**Figure 2**) due to the losses datasets being enriched also in zero-coverage genomic portions.

**Table 4:** Copy number variations statistics for the four analyzed Sardinian cultivars.

	Total				Private			
	#Gains	Gains (bp)	#Losses	Losses (bp)	#Gains	Gains (bp)	#Losses	Losses (bp)
Bovale	1104	9561500	571	4725000	694	3403500	482	2508000
Cannonau	2483	34328000	248	3252500	2252	27126000	178	1889500
Carignano	920	6224500	446	3475000	550	2769500	273	1511000
Vermentino	290	4017999	464	3718500	202	2322499	322	1920500

Transposable elements (TE) are known to play a primary role in shaping the genomic architecture of plants (Carrier et al., 2012; Bai et al., 2016) and may contribute to the occurrence of CNVs. Indeed a relevant, although variable, proportion of the detected CNV proved to overlap annotated TE for all the analyzed cultivars (Supplementary Table S3). We found that, on average, TE overlap 23.2 and 6.7% of the detected gains and losses, respectively. Notably, CNVs proved to host a higher percentage of TE in cultivar Cannonau with a relative abundance almost double than those observed for the other varieties. A more detailed analysis of TE types and distribution within CNV regions was beyond the scope of this manuscript and will be reported elsewhere.

The distribution of large deletions clearly differentiated the four Sardinian cultivars, with Cannonau featuring the highest number (1990), Vermentino the lowest (50) and Bovale and Carignano featuring intermediate numbers of 419 and 529, respectively (**Figure 2** and **Table 5**). Approximately 1,100,000 bp included in the large deletions was common to the four Sardinian varieties and this encompassed 44 protein-coding genes (Supplementary Table S2). The proportion of private large deletions ranged from 16.9% in Vermentino to 54.5% in Cannonau (**Table 5**).

**Table 5:** Large deletions statistics for the four analyzed Sardinian cultivars.

Cultivar	#Large deletions	Large deletions (bp)	Private (bp)	%Private
Bovale	412	26858904	7513499	28.0
Cannoanu	1990	156925773	85551167	54.5
Carignano	529	29768056	6171910	20.7
Vermentino	50	3779734	640538	16.9
Common	14	1195168		

### Functional Diversity

We next investigated whether the sequence and structural polymorphisms within genes provided insight into the adaptive and/or artificial selection traits of the cultivars. Gene ontology enrichment analysis was applied to the putative pseudogenes, revealing that several biological process categories such as “defense response” and “apoptotic process” were significantly overrepresented in the Sardinian cultivars (Supplementary Table S4).

We calculated the rate of non-synonymous (*d*n) and synonymous (*d*s) substitutions at loci featuring more than five SNPs and used the *d*n/*d*s ratio to identify the genes under either purifying (*d*n/*d*s < 1) or diversifying selection (*d*n/*d*s > 1). Gene ontology single-gene enrichment analysis revealed several common features among the Sardinian cultivars. Several genes involved in methionine biosynthesis appeared subject to purifying selection in all the red berry Sardinian varieties. Similarly, a number of genes involved in the regulation of auxin response factor (ARF) signal transduction appeared subject to purifying selection in all the Sardinian varieties with the exception of Carignano (Supplementary Table S5a). Finally, genes involved in apoptosis and other defense processes appeared subject to positive selection in all the cultivars (Supplementary Table S5b).

Gene ontology enrichment analysis of the genes within ROHs indicated the predominance of primary metabolism, stress response and secondary metabolism categories (Supplementary Table S6). However, within these wide classes each cultivar featured specific biological process or molecular functions. ROHs in Bovale were enriched for genes involved in defense responses and the biosynthesis of salicylic and jasmonic acids. ROHs in Cannonau were enriched for genes involved in solute transport across cellular membranes and responses to biotic and abiotic stress, such as cold, wounding and fungi. Stress response genes were also significantly enriched in the Carignano ROHs, together with genes encoding strictosidine synthetases and those involved in cytoskeletal organization. The ROHs in Vermentino were enriched for genes involved in embryo sac development, trehalose biosynthesis and oxidation/reduction.

The ontologies of genes in CNV regions depended on whether the regions were gains or losses. The gained regions were enriched for genes involved in flavonoid synthesis and other secondary metabolic processes, especially in Cannonau (Supplementary Table S7). In contrast, the lost regions were enriched for stress-response genes (Supplementary Table S8). As stated above, the gain or loss of regions was relative to the reference genome, so a significant enrichment should be interpreted as evidence that mutation (either deletion or duplication) has affected regions hosting specific gene functions rather than enrichment of the function with respect to the gene copy number in the reference sequence. Gene ontology enrichment analysis focusing on genes within large deletions also revealed the prevalence of genes that respond to biotic/abiotic stress. Notably, several ontologies were shared among the Sardinian cultivars, with 11 common genes involved in cycloartenol biosynthesis lost in three of the varieties (Supplementary Table S9).

## Discussion

Viticulture and wine-making play a primary role in the Sardinian economy. Indeed, almost 26,000 ha of the island is devoted to grapevine cultivation yielding ∼500,000 hectoliters of wine every year (Nieddu, 2011). Cannonau, Bovale and Carignano are among the most widespread red berry cultivars, and Vermentino is by far the most widely cultivated white berry cultivar. These varieties were resequenced to investigate genomic characteristics potentially associated with their distinct phenotypes.

### Genetic Diversity and Distribution of Sequence Polymorphisms

Sequence reads from the Sardinian varieties were aligned to the Pinot noir PN40024 reference genome, allowing the identification of several forms of sequence polymorphism, such as SNPs and indels, as well as structural variations such as CNVs, PAVs and large deletions. The cultivars showed wide variation in several sequence diversity parameters, and 2,421,176 SNPs were discovered by comparing the Sardinian cultivars with three varieties not grown in Sardinia.

Cannonau was most similar to the reference genome in terms of the percentage of homozygous SNPs and reference bases covered by reads (**Table 1**) whereas Vermentino showed the greatest divergence from the Pinot noir genome due to the greater number of genomic positions not covered by reads, and the frequency of SNPs/indels at both the genomic and genic levels. This may reflect the original selection of this cultivar for the production of table grapes (Nieddu, 2011), in accordance with previous studies highlighting marked genomic differences between wine and table varieties (Myles et al., 2011). Indeed, several alignment statistics were common between Vermentino and Sultanina, a well-known table variety, such as the high number of indels within the transcripts and the lower number of mutations producing stop codons (**Table 2**). The ratio of heterozygous to homozygous SNPs differed substantially among the cultivars, suggesting their breeding histories were also distinct. Bovale showed the lowest ratio of heterozygous to homozygous SNPs, and historical data suggest this cultivar originated by local breeding with the selection of several clones. Based on simple sequence repeat (SSR) polymorphism, several closely related clones have been identified that can be assigned to a cluster of Bovale-like genotypes (Meneghetti et al., 2013). The breeding of these clones may have been characterized by intercrossing and the selection of Bovale-related materials, including Bovale muristellu and Bovale murru (Grassi et al., 2003).

Uninterrupted arrays of homozygous SNPs, defined as ROHs, are often considered as signatures of inbreeding. Several ROH parameters are reliable predictors of the breeding histories of carriers, including their size, SNP density and distribution. Using a conservative approach, we identified the extent of ROHs in all the cultivars. Only a small proportion of the total ROH sequence was shared among all the cultivars. Notably, the length distributions of ROHs in each cultivar were similar, with most belonging to the smallest length classes. ROHs have been associated to inbreeding events in several systems and the length distributions of ROHs has been taken as a marker of the timing and extent of inbreeding: large ROHs are associated with recent inbreeding whereas smaller ones are older and thus usually diagnostic of germplasm origin. Following these considerations the rather homogenous ROH distributions we observed may reflect the limited number of sexual reproduction events typical of grape breeding. In Cannonau, the lower number of ROHs together with the higher percentage of heterozygous polymorphisms may suggests a more complex breeding history than the other cultivars grown in Sardinia. This is supported by evidence that Cannonau clusters more closely to varieties cultivated in the near East than with other Italian varieties (Crespan, 2010). However, we urge caution in interpreting these results only in terms of inbreeding because, in species vegetatively propagated, regions with reduced heterozygosity (and thus with high level of homozigosity) may be coincident with mosaic structural variations. Application of dedicated software together with resequencing experiments featuring higher depth of coverage will be needed to discriminate ROH origin in the analyzed cultivars (Marroni et al., 2017).

### Signatures of Selection

Selection for desirable traits may have driven the emergence of unique genomic features in each of the cultivars so we searched for genes under purifying and positive selection by calculating the *d*n/*d*s ratio at each polymorphic locus. Our data indicated that the selected traits play key roles in the plant life cycle. For example, six genes involved in methionine biosynthesis were found to be under purifying selection in all the Sardinian red berry varieties (Supplementary Table S5a). Methionine metabolism appears to be involved in the ripening of berries given that the derivative *S*-adenosylmethionine is required for the production of ethylene during maturation (Agudelo-Romero et al., 2013) and methionine precursors differ widely in abundance from veraison onward in diverse grapevine varieties (Giribaldi et al., 2010). Several ARF genes were also found to be under purifying selection, and this family of regulators is also implicated in grapevine berry ripening (Wan et al., 2014). Finally, an enrichment in biological processes involved in the cell shape regulation (e.g., “regulation of cell shape,” “microtubule-based movement,” “actin filament-based movement”) emerged when analyzing genes under purifying selection. In this regards the cytoskeleton of plant cells is believed to play a role in the response to several external stimuli such as heat or cold that are sensed as a mechanical load upon the membrane (Nick, 2013).

In contrast, stress-response genes (particularly those involved in apoptosis) were found to be under positive selection, which may provide the genetic variation needed to deal with a wider range of local conditions (Supplementary Table S5b). The plasticity of stress-response genes was also confirmed by the gene ontology enrichment analysis of transcripts featuring premature stop codons in all the cultivars. In this regard, previous reports highlighted a diverse response to abiotic stresses (e.g., water depletion) for cultivars Cannonau, Carignano and Bovale (Mercenaro et al., 2016a) with such a trend being also confirmed when Vermentino was compared to the international cv Chardonnay (Mercenaro et al., 2012). A wide variety of genes proved to be involved in the response to several abiotic stresses (e.g., high light, high heat and drought) also in other grapevine varieties. Interestingly, transcriptomic analyses revealed that the number and type of differentially expressed stress related genes may largely vary when comparing different cultivars resulting in candidate gene sets that are poorly overlapping (Rocheta et al., 2016).

### Structural Variation

Complex structural variations such as CNVs, PAVs and large deletions contribute to both intraspecies and interspecies genetic variation. CNV polymorphisms are widely studied in humans because they are associated with many severe diseases (Buchanan and Scherer, 2008). CNV has only recently been investigated in plants and CNVs may be more abundant in intergenic regions, although CNVs involving genes have also been reported (Żmieńko et al., 2013).

Copy number variation proved to be non-homogeneously distributed along the chromosomes of the analyzed cultivars. Interestingly, the occurrence of gains (and, at a lesser extent, losses) was not detected in extended portions of chromosomes 1, 10 and 17. Although we cannot exclude that technical reasons may have contributed to such a phenomenon (see Result), other causes should be taken into consideration. The presented varieties may share a higher homology with the reference PN40024 cultivar in the highlighted genomic portions. Indeed, chromosomes 1 and 17 featured SNPs frequency values below the average for all the analyzed cultivars (data not shown). Additionally, the unequal distribution of TE (whose presence is highly correlated with the occurrence of CNVs) may contribute to the observed trend (i.e., chromosome 17 contains 4.7 repetitive sequences per Mb, that is the lowest value among the *V. vinifera* chromosomes).

Having identified CNVs in the genomes of the four Sardinian cultivars, we extracted the associated genes and used a single-gene enrichment analysis to investigate their ontologies. We found that biological processes and molecular functions related to stress responses were the most overrepresented categories among these genes in all four cultivars (Supplementary Tables S7, S8). However, each cultivar was also characterized by unique ontologies. For example, among the molecular functions specifically overrepresented in the Cannonau gained genomic regions we found 13 genes involved in the synthesis of naringenin and resveratrol, and 6 of the 12 known genes involved in the synthesis of jasmonate, which enhances the production of resveratrol. This observation seems to be in line with previous reports highlighting a high content of this longevity-linked (Bhullar and Hubbard, 2015) secondary metabolite in Cannonau (Franco et al., 2000; Corder et al., 2006). Genes involved in the synthesis of resveratrol and naringenin-chalcone were also overrepresented in the gained regions of Bovale together with genes involved in the metabolism of hydrogen peroxide, e.g., a molecule whose accumulation proved to vary during the *V. vinifera* plant cycle (Qsaib et al., 2014). Notably, genes in the gained regions of Carignano and Vermentino shared several biological processes and molecular functions related to redox activity and electron transport.

The main processes represented by genes in the lost regions of all four cultivars were related to stress responses, thus confirming the widespread genomic plasticity of this class of genes. In Cannonau, the molecular function “chitinase activity” was also overrepresented in the lost regions, and this is associated with resistance to fungal pathogens (Busam et al., 1997). In Bovale, the molecular function “strictosidine synthase activity” was overrepresented in the lost regions, concurring that the absence of these enzymes in grapevine has no impact on fitness (Zhang et al., 2014).

Finally, several genes were lost in all the red berry varieties due to large deletions events. In particular, 11 genes with cycloartenol synthase activity were potentially lost in at least one allele of Cannonau, Bovale and Carignano. Cycloartenol synthase converts 2,3-oxidosqualene to cycloartenol, which is the first step in the biosynthesis of sterols. *Arabidopsis thaliana* plants with a mutation in this gene failed to produce progeny suggesting a role in male gametophyte function (Babiychuk et al., 2008). Because the grapevine cultivars we investigated have been bred by vegetative propagation for several centuries it is likely that some gene required for pollen development may be lost in large deletions without this phenomenon being counter selected. Notably a significantly higher number of ontologies associated with the synthesis of resveratrol emerged for genes within large deletions in Vermentino. This finding was confirmed in other white grape wines (Gewurztraminer and Sultanina), providing a genetic explanation for the lower resveratrol content of white wines compared to reds (Bavaresco, 2003).

## Conclusion

We produced a list of CNV, SNP and indels which could be of functional significance and thus contribute to explain agronomic differences among cultivars. Although the reported polymorphisms rely on a mere *in silico* investigation, the high stringency of the method together with an extensive quality check of our pipeline (see Data Sheet 1) allowed to produce reliable inferences. The integration of such data with transcriptomic and metabolomic analyses under different stress conditions will allow to narrow the number of candidate regions under investigations and construct hypothesis breeding strategies to improve *V. vinifera* resilience.

## Author Contributions

SC and AP contributed to the design and conception of the work together to the drafting of the manuscript. LM, GN, and MP contributed to the interpretation of the generated data and to the revision of the manuscript. All the authors approved the final draft of the submitted manuscript.

## Conflict of Interest Statement

The authors declare that the research was conducted in the absence of any commercial or financial relationships that could be construed as a potential conflict of interest.

## Footnotes

1 http://www.oiv.int/

2 http://genomes.cribi.unipd.it/grape/

3 https://github.com/nh13/DWGSIM

## Supplementary Material

FIGURE S1 Coverage distribution along 19 chromosomes of *Vitis vinifera* for the 4 cultivars grown in Sardinia. All possible mapping locations were considered at this stage in order to highlight common patterns due to highly repetitive regions. From outward to inward: 19 *Vitis vinifera* chromosomes ideogram, Bovale, Cannonau, Carignano, Vermentino. Colors range ascendingly from green (low coverage) to red (high coverage) and represents coverage values that were normalized on the individual average genomic coverage (9 color classes were used, e.g., green, dgreen, vdgreen, vvdgreen, black, vvdred, vdred, dred, red, with color prefixes v, very and d, dark).

FIGURE S2 Length distribution of gained regions, lost regions and large deletions, in the four analyzed Sardinian grape cultivars.
