# Supplementary figures and images for: Sequence Polymorphisms and Structural Variations among Four Grapevine (Vitis vinifera L.) Cultivars Representing Sardinian Agriculture
Source: Front Plant Sci. 2017 Jul 20;8:1279. doi: 10.3389/fpls.2017.01279 (PMC5517397; doi:10.3389/fpls.2017.01279)

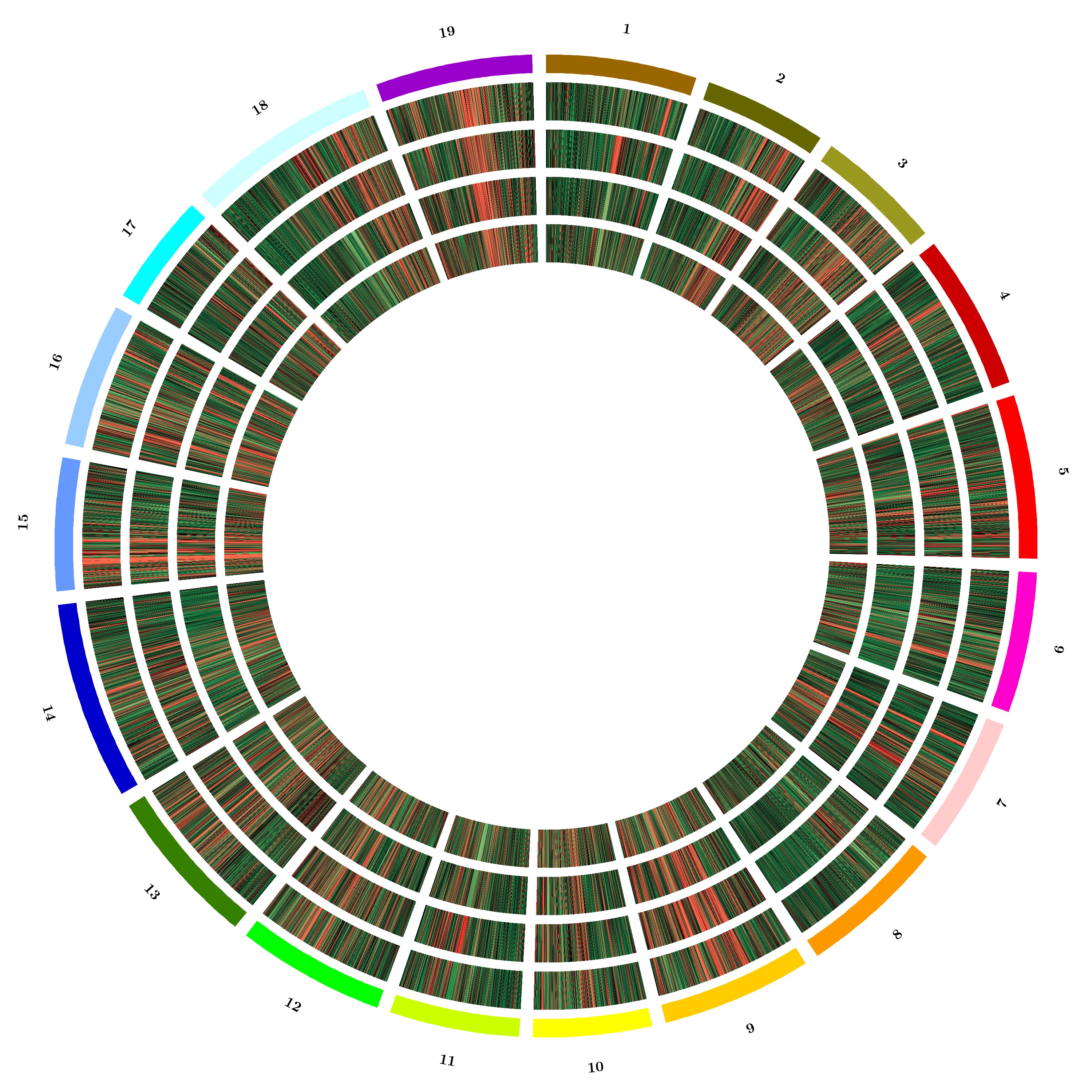

Supplement: FIGURE S1 — Coverage distribution along 19 chromosomes of Vitis vinifera for the 4 cultivars grown in Sardinia. All possible mapping locations were considered at this stage in order to highlight common patterns due to highly repetitive regions. From outward to inward: 19 Vitis vinifera chromosomes ideogram, Bovale, Cannonau, Carignano, Vermentino. Colors range ascendingly from green (low coverage) to red (high coverage) and represents coverage values that were normalized on the individual average genomic coverage (9 color classes were used, e.g., green, dgreen, vdgreen, vvdgreen, black, vvdred, vdred, dred, red, with color prefixes v, very and d, dark). [file Image_1.JPEG]

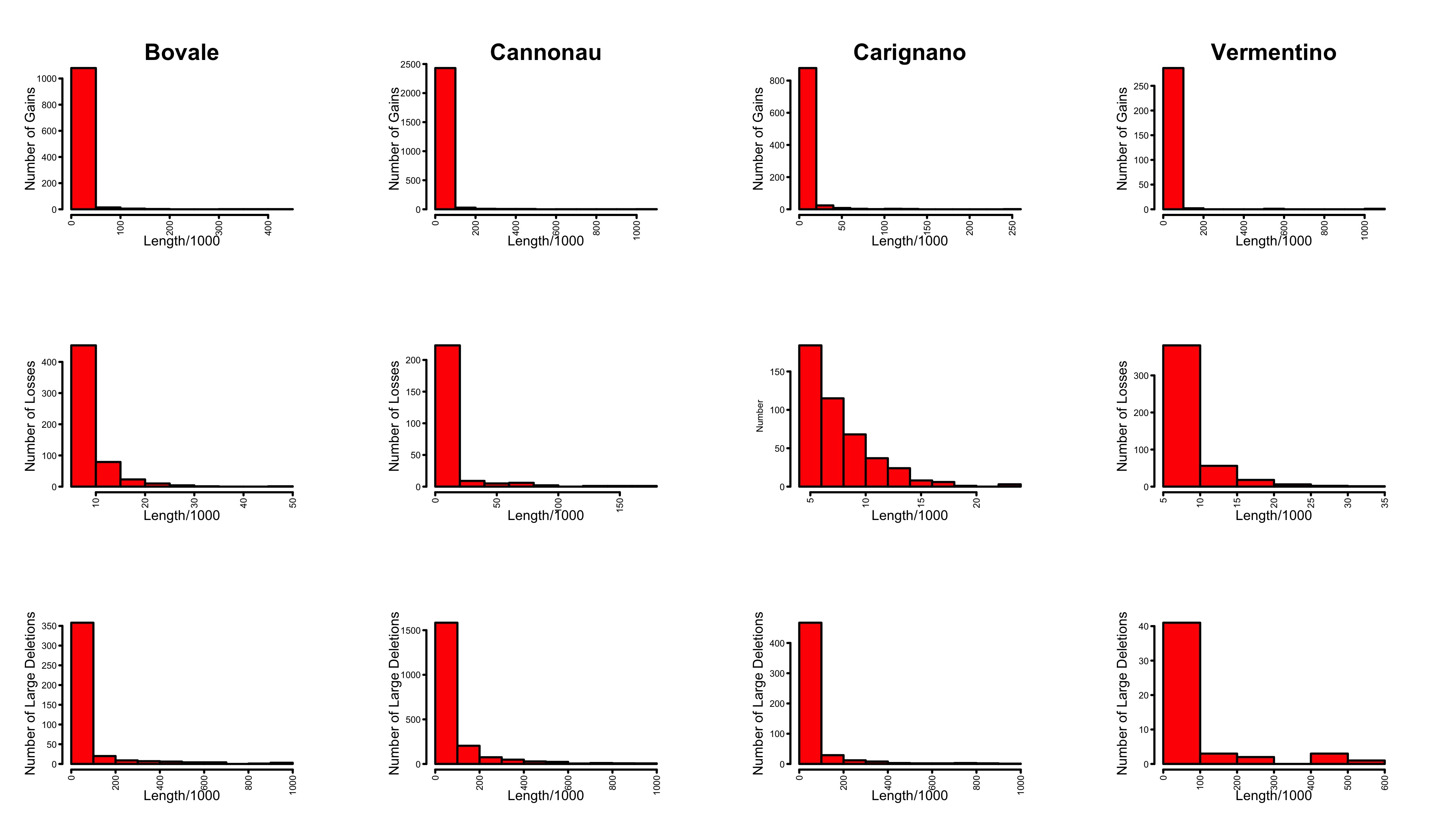

Supplement: FIGURE S2 — Length distribution of gained regions, lost regions and large deletions, in the four analyzed Sardinian grape cultivars. [file Image_2.JPEG]
