# Supplementary material for: Sequence Polymorphisms and Structural Variations among Four Grapevine (Vitis vinifera L.) Cultivars Representing Sardinian Agriculture
Source: Front Plant Sci. 2017 Jul 20;8:1279. doi: 10.3389/fpls.2017.01279 (PMC5517397; doi:10.3389/fpls.2017.01279)
Supplement: Supplementary file 3 [file Table_1.DOCX]

**Table S1:** Resequencing statistics for the 4 analysed grapevine cultivars

| **Variety** | **Reads length(bp)** | **Number of bases in HQ reads** |
| --- | --- | --- |
| Bovale | 101 | 3,403,252,570 |
| Cannonau | 75 -113 | 13,015,022,612 |
| Carignano | 101 | 5,801,933,082 |
| Vermentino | 101 | 6,477,411,588 |
| Gewurztraminer (ERR514999) | 100 | 16,406,114,754 |
| Sultanina (SRR924200) | 101 | 56,374,684,661 |
| Tannat (SRR863618) | 101 | 40,884,163,618 |
| Tannat (SRR863595) | 101 | 16,328,290,150 |
