# Supplementary material for: Sequence Polymorphisms and Structural Variations among Four Grapevine (Vitis vinifera L.) Cultivars Representing Sardinian Agriculture
Source: Front Plant Sci. 2017 Jul 20;8:1279. doi: 10.3389/fpls.2017.01279 (PMC5517397; doi:10.3389/fpls.2017.01279)
Supplement: Supplementary file 4 [file Table_2.DOCX]

**Table S2:** Genes within the structural variations (gains, losses and large deletions) and run of homozygosity regions that are common to the four analysed Sardinian grape cultivars.

| Gains | Losses | Large deletions | ROH |
| --- | --- | --- | --- |
| VIT_13s0067g01850 | VIT_16s0022g02500 | VIT_01s0010g01280 | VIT_03s0017g02230 |
| VIT_13s0101g00220 | VIT_16s0100g00150 | VIT_01s0010g01290 | VIT_06s0009g00450 |
| VIT_13s0101g00230 | VIT_16s0022g02520 | VIT_01s0010g01310 | VIT_06s0009g00720 |
| VIT_15s0024g01230 | VIT_16s0022g02530 | VIT_01s0010g01320 | VIT_10s0042g00290 |
| VIT_15s0045g00050 | VIT_10s0092g00500 | VIT_01s0010g01330 | VIT_10s0042g00410 |
| VIT_08s0056g01050 | VIT_07s0151g00390 | VIT_01s0010g01340 | VIT_11s0016g01310 |
|  |  | VIT_01s0010g01350 | VIT_12s0034g00690 |
|  |  | VIT_01s0010g01360 | VIT_12s0034g00700 |
|  |  | VIT_01s0010g01380 | VIT_12s0035g01540 |
|  |  | VIT_12s0035g00430 | VIT_13s0101g00290 |
|  |  | VIT_12s0035g00440 | VIT_13s0101g00300 |
|  |  | VIT_12s0035g00450 | VIT_13s0101g00320 |
|  |  | VIT_12s0035g00460 | VIT_13s0101g00330 |
|  |  | VIT_12s0035g00470 | VIT_13s0047g00740 |
|  |  | VIT_12s0035g00480 | VIT_13s0047g01000 |
|  |  | VIT_12s0035g00490 | VIT_14s0083g00220 |
|  |  | VIT_13s0047g00830 | VIT_14s0083g00230 |
|  |  | VIT_13s0047g00840 | VIT_14s0083g00240 |
|  |  | VIT_13s0047g00850 | VIT_16s0022g00340 |
|  |  | VIT_13s0047g00860 | VIT_16s0022g00350 |
|  |  | VIT_13s0047g00870 | VIT_16s0022g00360 |
|  |  | VIT_13s0047g00890 | VIT_16s0022g00840 |
|  |  | VIT_13s0047g00910 | VIT_16s0022g00850 |
|  |  | VIT_13s0047g00920 | VIT_16s0022g01330 |
|  |  | VIT_13s0047g00940 | VIT_16s0022g01430 |
|  |  | VIT_13s0047g00950 | VIT_16s0100g01160 |
|  |  | VIT_13s0047g00960 | VIT_18s0086g00560 |
|  |  | VIT_09s0070g00800 | VIT_19s0027g01180 |
|  |  | VIT_09s0070g00810 | VIT_19s0027g01480 |
|  |  | VIT_09s0070g00820 | VIT_19s0027g01570 |
|  |  | VIT_09s0070g00840 | VIT_19s0027g01560 |
|  |  | VIT_09s0070g00850 |  |
|  |  | VIT_09s0070g00860 |  |
|  |  | VIT_09s0070g00870 |  |
|  |  | VIT_09s0070g00880 |  |
|  |  | VIT_09s0070g00890 |  |
|  |  | VIT_09s0070g00900 |  |
|  |  | VIT_09s0070g00920 |  |
|  |  | VIT_09s0070g00940 |  |
|  |  | VIT_09s0070g00950 |  |
|  |  | VIT_09s0070g00960 |  |
|  |  | VIT_09s0070g00970 |  |
|  |  | VIT_09s0070g00980 |  |
|  |  | VIT_09s0070g01030 |  |
