# Supplementary material for: Sequence Polymorphisms and Structural Variations among Four Grapevine (Vitis vinifera L.) Cultivars Representing Sardinian Agriculture
Source: Front Plant Sci. 2017 Jul 20;8:1279. doi: 10.3389/fpls.2017.01279 (PMC5517397; doi:10.3389/fpls.2017.01279)
Supplement: Supplementary file 5 [file Table_3.DOCX]

**Table S3:** Number of repetitive regions overlapping genomic portions involved in copy number variations. Percentages relative to the total number of gains/losses are reported between brackets.

| **Cultivar** | **Repeats in gains** | **Repeats in losses** |
| --- | --- | --- |
| Bovale | 209(18.9%) | 32 (5.6%) |
| Cannonau | 867 (34.9%) | 25 (10.1%) |
| Carignano | 192 (20.9%) | 28 (6.3%) |
| Vermentino | 53 (18.3%) | 23 (5.0%) |
