# Supplementary material for: Sequence Polymorphisms and Structural Variations among Four Grapevine (Vitis vinifera L.) Cultivars Representing Sardinian Agriculture
Source: Front Plant Sci. 2017 Jul 20;8:1279. doi: 10.3389/fpls.2017.01279 (PMC5517397; doi:10.3389/fpls.2017.01279)
Supplement: Supplementary file 6 [file Table_4.DOCX]

**Table S4:** Gene ontology Single gene enrichment analysis of transcripts with premature stop codons. In brackets two numbers are reported representing the number of occurrences of the reported ontology in the universal dataset and in the analysed gene set respectively (p < 0.05).

| **Cultivar** | **BP** | **MF** |
| --- | --- | --- |
| **Bovale** | apoptotic process (501/13) | ATP binding(3468/47) |
|  | defense response(812/16) | nucleoside-triphosphatase activity(1167/20) |
|  | protein phosphorylation(1563/21) | anion binding(4318/59) |
|  | tRNA methylation(1/1) | protein binding(4676/47) |
|  |  | protein serine/threonine kinase activity(1427/19) |
|  |  | 8-methylthiopropyl glucosinolate S-oxyge...(1/1) |
|  |  | tRNA (adenine-N1-)-methyltransferase act...(1/1) |
|  |  | motor activity(114/4) |
|  |  |  |
| **Cannonau** | apoptotic process(501/48) | ATP binding(3468/203) |
|  | protein phosphorylation(1563/85) | protein serine/threonine kinase activity(1427/81) |
|  | defense response(812/49) | 2-alkenal reductase [NAD(P)] activity(400/32) |
|  | oxidation-reduction process(2476/102) | receptor activity(1309/64) |
|  |  | protein binding(4676/178) |
|  |  | biotin binding(7/3) |
|  |  | nucleoside-triphosphatase activity(1167/61) |
|  |  | flavin adenine dinucleotide binding(175/13) |
|  |  | non-membrane spanning protein tyrosine k...(179/13) |
|  |  | xenobiotic-transporting ATPase activity(50/6) |
|  |  |  |
| **Carignano** | apoptotic process(501/23) | ATP binding(3468/84) |
|  | protein phosphorylation(1563/39) | protein serine/threonine kinase activity(1427/38) |
|  | defense response(812/24) | 2-alkenal reductase [NAD(P)] activity(400/15) |
|  | recognition of pollen(184/9) | protein binding(4676/78) |
|  | autophagy(27/3) | receptor activity(1309/34) |
|  | calcium-mediated signaling(31/3) | (+)-delta-cadinene synthase activity(58/5) |
|  | G-protein coupled receptor signaling pat...(72/4) | nucleoside-triphosphatase activity(1167/26) |
|  |  | ionotropic glutamate receptor activity(38/4) |
|  |  | extracellular-glutamate-gated ion channe...(38/4) |
|  |  |  |
| **Vermentino** | apoptotic process(501/13) | ATP binding(3468/46) |
|  | innate immune response(147/7) | transmembrane signaling receptor activit...(167/7) |
|  | signal transduction(813/14) | biotin binding(7/2) |
|  | phosphorylation(1686/22) | nucleoside-triphosphatase activity(1167/17) |
|  | resolution of meiotic recombination inte...(1/1) | magnesium ion binding(229/6) |
|  | wax biosynthetic process(18/2) | 8-methylthiopropyl glucosinolate S-oxyge...(1/1) |
|  |  | protein serine/threonine kinase activity(1427/19) |
