# Supplementary material for: Sequence Polymorphisms and Structural Variations among Four Grapevine (Vitis vinifera L.) Cultivars Representing Sardinian Agriculture
Source: Front Plant Sci. 2017 Jul 20;8:1279. doi: 10.3389/fpls.2017.01279 (PMC5517397; doi:10.3389/fpls.2017.01279)
Supplement: Supplementary file 7 [file Table_5.DOCX]

**Table S5a:** Gene ontology Single gene enrichment analysis of transcript under purifying selection. In brackets two numbers are reported representing the number of occurrences of the reported ontology in the universal dataset and in the analysed gene set respectively (p < 0.05).

| **Cultivar** | **BP** | **MF** |
| --- | --- | --- |
| **Bovale** | porphyrin-containing compound biosynthet...(32/10) | ribonucleoside binding(3727/269) |
|  | regulation of ARF protein signal transdu...(5/4) | nucleoside binding(3728/269) |
|  | methionine biosynthetic process(21/6) | ATP binding(3468/251) |
|  | proteolysis(826/66) | hydrolase activity(3921/278) |
|  | single-organism cellular process(5212/340) | adenyl ribonucleotide binding(3481/251) |
|  | microtubule-based movement(110/14) | adenyl nucleotide binding(3484/251) |
|  | negative regulation of catalytic activit...(88/12) | purine ribonucleoside triphosphate bindi...(3717/265) |
|  | regulation of protein localization(7/3) | protein binding(4676/322) |
|  | cellular component organization(844/63) |  |
|  | pigment biosynthetic process(52/8) |  |
|  |  |  |
| **Cannonau** | methionine biosynthetic process(21/6) | ATP binding(3468/245) |
|  | transmembrane transport(849/67) | calcium-transporting ATPase activity(29/8) |
|  | calcium ion transport(60/10) | 2-alkenal reductase [NAD(P)] activity(400/38) |
|  | regulation of ARF protein signal transdu...(5/3) | organic phosphonate transmembrane-transp...(55/10) |
|  | chromatin modification(115/14) | catalytic activity(11576/705) |
|  | peptidyl-histidine modification(21/5) | 4-coumarate-CoA ligase activity(15/5) |
|  | phosphate-containing compound metabolic ...(2194/142) | ARF guanyl-nucleotide exchange factor ac...(5/3) |
|  |  | L-iditol 2-dehydrogenase activity(2/2) |
|  |  | nucleoside-triphosphatase activity(1167/100) |
|  |  | xylanase activity(20/5) |
|  |  |  |
| **Carignano** | negative regulation of catalytic activit...(88/16) | ATP binding(3468/268) |
|  | actin filament-based movement(10/5) | 2-alkenal reductase [NAD(P)] activity(400/49) |
|  | protein metabolic process(3736/273) | identical protein binding(115/19) |
|  | methionine biosynthetic process(21/6) | serine-type endopeptidase activity(175/24) |
|  | cellular aldehyde metabolic process(48/12) | L-malate dehydrogenase activity(12/5) |
|  | divalent metal ion transport(81/12) | organic phosphonate transmembrane-transp...(55/10) |
|  | starch metabolic process(11/4) | protein binding(4676/321) |
|  | glyoxylate cycle(11/4) | disaccharide transmembrane transporter a...(5/3) |
|  | peptidyl-histidine phosphorylation(18/5) | divalent inorganic cation transmembrane ...(80/12) |
|  |  | pectate lyase activity(17/5) |
|  |  |  |
| **Vermentino** | regulation of cell shape(12/5) | ATP binding(3468/264) |
|  | microtubule-based movement(110/15) | MAP kinase kinase kinase activity(86/20) |
|  | plant-type spore development(5/3) | 2-alkenal reductase [NAD(P)] activity(400/45) |
|  | regulation of ARF protein signal transdu...(5/3) | protein binding(4676/311) |
|  | single-organism cellular process(5212/351) | beta-galactosidase activity(18/7) |
|  | manganese ion transport(6/3) | serine-type endopeptidase activity(175/21) |
|  | single-organism biosynthetic process(1140/84) | motor activity(114/15) |
|  | telomere maintenance(2/2) | xenobiotic-transporting ATPase activity(50/9) |
|  | protein phosphorylation(1563/110) | protein xylosyltransferase activity(10/4) |
|  |  | ARF guanyl-nucleotide exchange factor ac...(5/3) |

**Table S5b:** Gene ontology Single gene enrichment analysis of transcript under positive selection. In brackets two numbers are reported representing the number of occurrences of the reported ontology in the universal dataset and in the analysed gene set respectively (p < 0.05).

| **Cultivar** | **BP** | **MF** |
| --- | --- | --- |
| **Bovale** | apoptotic process(501/11) | ATP binding(3468/23) |
|  | defense response(812/10) | protein binding(4676/27) |
|  |  | phosphoprotein phosphatase activity(340/5) |
|  |  |  |
| **Cannonau** | apoptotic process(501/19) | ATP binding(3468/35) |
|  | defense response(812/22) | licheninase activity(10/2) |
|  |  | glucan endo-1,3-beta-D-glucosidase activ...(43/3) |
|  |  | nucleoside-triphosphatase activity(1167/12) |
|  |  | cinnamoyl-CoA reductase activity(34/2) |
|  |  |  |
| **Carignano** | apoptotic process(501/10) | licheninase activity(10/2) |
|  | defense response(812/12) | ATP binding(3468/23) |
|  |  | receptor activity(1309/12) |
|  |  | catalytic activity(11576/55) |
|  |  | protein tyrosine kinase activity(307/5) |
|  |  |  |
| **Vermentino** | apoptotic process(501/15) | ATP binding(3468/28) |
|  | defense response(812/14) | xanthine oxidase activity(1/1) |
|  | innate immune response(147/4) | transmembrane signaling receptor activit...(167/4) |
|  | tyrosine catabolic process(2/1) | xanthine dehydrogenase activity(2/1) |
