# Supplementary material for: Sequence Polymorphisms and Structural Variations among Four Grapevine (Vitis vinifera L.) Cultivars Representing Sardinian Agriculture
Source: Front Plant Sci. 2017 Jul 20;8:1279. doi: 10.3389/fpls.2017.01279 (PMC5517397; doi:10.3389/fpls.2017.01279)
Supplement: Supplementary file 8 [file Table_6.DOCX]

**Table S6:** Gene ontology Single gene enrichment analysis of transcripts included in run of homozygosity. In brackets two numbers are reported representing the number of occurrences of the reported ontology in the universal dataset and in the analysed gene set respectively (p < 0.05).

| **Cultivar** | **BP** | **MF** |
| --- | --- | --- |
| **Bovale** | apoptotic process(501/107) | heme binding(565/111) |
|  | defense response(812/145) | ATP binding(3468/501) |
|  | positive regulation of cell death(4/4) | strictosidine synthase activity(34/12) |
|  | salicylic acid mediated signaling pathwa...(4/4) | prunasin beta-glucosidase activity(26/10) |
|  | regulation of jasmonic acid biosynthetic...(4/4) | phenylalanine-tRNA ligase activity(22/9) |
|  | regulation of salicylic acid biosyntheti...(4/4) | long-chain-alcohol O-fatty-acyltransfera...(16/7) |
|  | protein phosphorylation(1563/231) | monooxygenase activity(510/91) |
|  |  | adenosylmethionine decarboxylase activit...(6/4) |
|  |  | chlorophyllase activity(6/4) |
|  |  |  |
| **Cannonau** | auxin-activated signaling pathway(134/27) | prephenate dehydrogenase (NADP+) activit...(6/3) |
|  | response to cold(102/16) | polygalacturonase activity(64/8) |
|  | defense response to fungus, incompatible...(4/3) | enzyme inhibitor activity(128/12) |
|  | metal ion transport(330/27) | CDP-alcohol phosphatidyltransferase acti...(3/2) |
|  | response to wounding(26/6) | calcium-transporting ATPase activity(29/5) |
|  | tyrosine biosynthetic process(6/3) | ubiquitin protein ligase binding(11/3) |
|  | L-methionine biosynthetic process from m...(6/3) | glutaminyl-tRNA synthase (glutamine-hydr...(4/2) |
|  | spermidine biosynthetic process(13/4) |  |
|  |  |  |
|  |  |  |
| **Carignano** | defense response(812/116) | strictosidine synthase activity(34/14) |
|  | negative regulation of catalytic activit...(88/22) | electron carrier activity(651/92) |
|  | response to biotic stimulus(223/41) | pyridoxine:NADP 4-dehydrogenase activity(12/7) |
|  | defense response, incompatible interacti...(29/9) | heme binding(565/74) |
|  | steroid biosynthetic process(74/16) | identical protein binding(115/22) |
|  | iron-sulfur cluster assembly(18/6) | flavonoid 3',5'-hydroxylase activity(97/18) |
|  | response to salicylic acid(43/10) | serine-type carboxypeptidase activity(61/13) |
|  | salicylic acid metabolic process(19/6) | phosphoprotein phosphatase activity(340/45) |
|  | microtubule cytoskeleton organization(25/7) | adenylylsulfate kinase activity(4/3) |
|  | protein transport(441/56) | glucan endo-1,3-beta-D-glucosidase activ...(43/10) |
| **Vermentino** | trehalose biosynthetic process(21/7) | nitrilase activity(11/7) |
|  | oxidation-reduction process(2476/237) | flavonol synthase activity(62/16) |
|  | glycerol-3-phosphate metabolic process(8/4) | glycerol kinase activity(3/3) |
|  | 7-methylguanosine mRNA capping(9/4) | chlorophyllase activity(6/4) |
|  | nucleosome assembly(72/13) | oxidoreductase activity, acting on singl...(193/29) |
|  | embryo sac development(21/6) | 3-beta-hydroxy-delta5-steroid dehydrogen...(48/10) |
|  |  | sterol 14-demethylase activity(2/2) |
|  |  | nitrilase activity(11/7) |
|  |  | flavonol synthase activity(62/16) |
|  |  |  |
