# Supplementary material for: Sequence Polymorphisms and Structural Variations among Four Grapevine (Vitis vinifera L.) Cultivars Representing Sardinian Agriculture
Source: Front Plant Sci. 2017 Jul 20;8:1279. doi: 10.3389/fpls.2017.01279 (PMC5517397; doi:10.3389/fpls.2017.01279)
Supplement: Supplementary file 9 [file Table_7.DOCX]

**Table S7:** Gene ontology Single gene enrichment analysis of transcript within gained portions. In brackets two numbers are reported representing the number of occurrences of the reported ontology in the universal dataset and in the analysed gene set respectively (p < 0.05).

| **Cultivar** | **BP** | **MF** |
| --- | --- | --- |
| **Bovale** | flavonoid biosynthetic process(76/7) | manganese ion binding(87/10) |
|  | ATP synthesis coupled electron transport(16/3) | superoxide dismutase activity(81/9) |
|  | lignin catabolic process(75/5) | trihydroxystilbene synthase activity(42/7) |
|  | respiratory chain complex IV assembly(7/2) | naringenin-chalcone synthase activity(45/7) |
|  | photosynthesis, light reaction(59/4) | nutrient reservoir activity(120/9) |
|  | protein N-linked glycosylation(9/2) | NADH dehydrogenase (ubiquinone) activity(32/5) |
|  |  | quinone binding(35/5) |
|  |  | alpha-1,3-mannosylglycoprotein 2-beta-N-...(3/2) |
|  |  | DNA-directed RNA polymerase activity(75/5) |
|  |  | receptor activity(1309/24) |
|  |  |  |
|  |  |  |
| **Cannonau** | apoptotic process(501/35) | trihydroxystilbene synthase activity(42/13) |
|  | flavonoid biosynthetic process(76/13) | naringenin-chalcone synthase activity(45/13) |
|  | defense response(812/36) | 12-oxophytodienoate reductase activity(12/6) |
|  | oligopeptide transport(76/6) | cycloartenol synthase activity(31/8) |
|  | cysteinyl-tRNA aminoacylation(6/2) | ATP binding(3468/118) |
|  |  | nucleoside-triphosphatase activity(1167/51) |
|  |  | tRNA (guanine-N2-)-methyltransferase act...(7/3) |
|  |  | structural constituent of nuclear pore(7/3) |
|  |  | superoxide dismutase activity(81/8) |
|  |  |  |
| **Carignano** | lignin catabolic process(75/6) | quinone binding(35/7) |
|  | ATP synthesis coupled electron transport(16/3) | copper ion binding(206/11) |
|  | respiratory chain complex IV assembly(7/2) | L-ascorbate oxidase activity(88/7) |
|  | sucrose metabolic process(15/2) | NADH dehydrogenase (ubiquinone) activity(32/5) |
|  | photosynthetic electron transport chain(21/2) | cycloartenol synthase activity(31/4) |
|  | protein kinase C-activating G-protein co...(23/2) | sucrose synthase activity(7/2) |
|  |  | tRNA (guanine-N2-)-methyltransferase act...(7/2) |
|  |  | extracellular-glutamate-gated ion channe...(38/3) |
|  |  |  |
| **Vermentino** | cell wall modification(77/6) | quinone binding(35/6) |
|  | photosynthesis, light reaction(59/5) | pectinesterase activity(83/7) |
|  | respiratory chain complex IV assembly(7/2) | aspartyl esterase activity(57/6) |
|  | regulation of pH(7/2) | enzyme inhibitor activity(128/7) |
|  | oxidation-reduction process(2476/33) | copper ion binding(206/7) |
|  | protein-chromophore linkage(35/3) | chlorophyll binding(25/3) |
|  | lignin catabolic process(75/4) | flavonoid 3'-monooxygenase activity(61/4) |
|  | ATP synthesis coupled electron transport(16/2) | sodium:proton antiporter activity(7/2) |
|  |  | NADH dehydrogenase (ubiquinone) activity(32/3) |
|  |  | strictosidine synthase activity(34/3) |
