# Supplementary material for: Sequence Polymorphisms and Structural Variations among Four Grapevine (Vitis vinifera L.) Cultivars Representing Sardinian Agriculture
Source: Front Plant Sci. 2017 Jul 20;8:1279. doi: 10.3389/fpls.2017.01279 (PMC5517397; doi:10.3389/fpls.2017.01279)
Supplement: Supplementary file 10 [file Table_8.DOCX]

**Table S8:** Gene ontology Single gene enrichment analysis of transcript within losses portions. In brackets two numbers are reported representing the number of occurrences of the reported ontology in the universal dataset and in the analysed gene set respectively (p < 0.05).

| **Cultivar** | **BP** | **MF** |
| --- | --- | --- |
| **Bovale** | protein phosphorylation(1563/26) | strictosidine synthase activity(34/7) |
|  | protein polymerization(61/4) | thiamine pyrophosphate binding(27/4) |
|  | microtubule-based movement(110/5) | GTP binding(282/9) |
|  |  | structural molecule activity(522/13) |
|  |  | protein kinase activity(1606/26) |
|  |  | acyl-[acyl-carrier-protein] desaturase a...(15/2) |
|  |  |  |
| **Cannonau** | killing of cells of other organism(21/10) | chitinase activity(58/11) |
|  | defense response to fungus(52/13) | chitin binding(29/8) |
|  | chitin catabolic process(53/11) | N,N-dimethylaniline monooxygenase activi...(35/6) |
|  | ethylene-activated signaling pathway(84/12) | transcription factor activity, sequence-...(548/25) |
|  | defense response(812/50) | tyramine N-feruloyltransferase activity(2/2) |
|  | cell wall macromolecule catabolic proces...(58/10) | phosphoprotein phosphatase activity(340/16) |
|  | response to desiccation(5/3) | NADP binding(63/6) |
|  | response to karrikin(41/6) | 3-beta-hydroxy-delta5-steroid dehydrogen...(48/5) |
|  | anaerobic respiration(8/3) | DNA binding(2441/65) |
|  | apoptotic process(501/20) | hydroxymethylglutaryl-CoA synthase activ...(7/2) |
|  |  |  |
| **Carignano** | innate immune response(147/11) | transmembrane signaling receptor activit...(167/9) |
|  | apoptotic process(501/19) | ATP binding(3468/67) |
|  | ethylene-activated signaling pathway(84/7) |  |
|  | defense response(812/34) |  |
|  | defense response to other organism(109/6) |  |
|  | response to virus(31/3) |  |
|  |  |  |
| **Vermentino** | protein phosphorylation(1563/26) | ATP binding(3468/48) |
|  | cysteinyl-tRNA aminoacylation(6/2) | cysteine-tRNA ligase activity(6/2) |
|  | apoptotic process(501/10) | salutaridinol 7-O-acetyltransferase acti...(9/2) |
|  | innate immune response(147/5) | transmembrane signaling receptor activit...(167/6) |
|  | DNA-templated transcription, elongation(16/2) | N,N-dimethylaniline monooxygenase activi...(35/3) |
|  |  | protein serine/threonine kinase activity(1427/22) |
|  |  | GTP binding(282/7) |
