# Supplementary material for: Sequence Polymorphisms and Structural Variations among Four Grapevine (Vitis vinifera L.) Cultivars Representing Sardinian Agriculture
Source: Front Plant Sci. 2017 Jul 20;8:1279. doi: 10.3389/fpls.2017.01279 (PMC5517397; doi:10.3389/fpls.2017.01279)
Supplement: Supplementary file 11 [file Table_9.DOCX]

**Table S9:** Gene ontology Single gene enrichment analysis of transcript within large deletions. In brackets two numbers are reported representing the number of occurrences of the reported ontology in the universal dataset and in the analysed gene set respectively (p < 0.05).

| **Cultivar** | **BP** | **MF** |
| --- | --- | --- |
| **Bovale** | oligopeptide transport(76/11) | cycloartenol synthase activity(31/12) |
|  | xylan catabolic process(20/6) | sinapate 1-glucosyltransferase activity(28/11) |
|  | chitin catabolic process(53/8) | limonoid glucosyltransferase activity(36/11) |
|  | response to desiccation(5/3) | transferase activity, transferring hexos...(578/53) |
|  | defense response(812/43) | lysozyme activity(14/7) |
|  | apoptotic process(501/30) | indole-3-acetate beta-glucosyltransferas...(36/10) |
|  | toxin catabolic process(78/9) | (iso)eugenol O-methyltransferase activit...(28/9) |
|  | oxygen transport(10/3) | chitinase activity(58/8) |
|  | L-phenylalanine catabolic process(21/4) | ammonia-lyase activity(22/5) |
|  | sucrose transport(5/2) | ATP binding(3468/135) |
|  |  |  |
| **Cannoanu** | apoptotic process(501/44) | cycloartenol synthase activity(31/12) |
|  | defense response(812/45) | nucleoside-triphosphatase activity(1167/58) |
|  | carbohydrate transport(105/13) | ATP binding(3468/109) |
|  | oxidation-reduction process(2476/75) | 2-alkenal reductase [NAD(P)] activity(400/26) |
|  | calcium ion transport(60/5) | protein tyrosine kinase activity(307/18) |
|  | glycerol-3-phosphate metabolic process(8/2) | organic phosphonate transmembrane-transp...(55/7) |
|  |  | heme binding(565/23) |
|  |  | phosphoprotein phosphatase activity(340/16) |
|  |  | ionotropic glutamate receptor activity(38/5) |
|  |  |  |
| **Carignano** | chitin catabolic process(53/9) | cycloartenol synthase activity(31/10) |
|  | xylan catabolic process(20/6) | lysozyme activity(14/7) |
|  | apoptotic process(501/24) | 12-oxophytodienoate reductase activity(12/6) |
|  | response to desiccation(5/3) | hydrogen-translocating pyrophosphatase a...(9/5) |
|  | superoxide metabolic process(17/4) | chitinase activity(58/9) |
|  | lipid catabolic process(155/10) | FMN binding(45/7) |
|  | defense response(812/26) | phosphoprotein phosphatase activity(340/18) |
|  | tyrosine biosynthetic process(6/2) | dihydrolipoyl dehydrogenase activity(6/3) |
|  |  | inorganic diphosphatase activity(33/5) |
|  |  | protein tyrosine kinase activity(307/15) |
|  |  |  |
| **Vermentino** | flavonoid biosynthetic process(76/4) | trihydroxystilbene synthase activity(42/4) |
|  | telomere maintenance(2/1) | naringenin-chalcone synthase activity(45/4) |
|  |  | transcription cofactor activity(41/3) |
|  |  | structural constituent of cell wall(17/2) |
|  |  | protein tyrosine kinase activity(307/4) |
|  |  | phosphoethanolamine N-methyltransferase ...(3/1) |
|  |  | aldose 1-epimerase activity(4/1) |
