# Supplementary material for: Sequence Polymorphisms and Structural Variations among Four Grapevine (Vitis vinifera L.) Cultivars Representing Sardinian Agriculture
Source: Front Plant Sci. 2017 Jul 20;8:1279. doi: 10.3389/fpls.2017.01279 (PMC5517397; doi:10.3389/fpls.2017.01279)
Supplement: Supplementary file 12 [file Data_Sheet_1.DOCX]

**Pipeline quality check**

**Filtering of high quality reads/bases**

All the produced reads were quality filter by using the software NGS QC Toolkit with default parameters. Only reads with average quality > 25 were retained for downstream analyses (Figure A1).


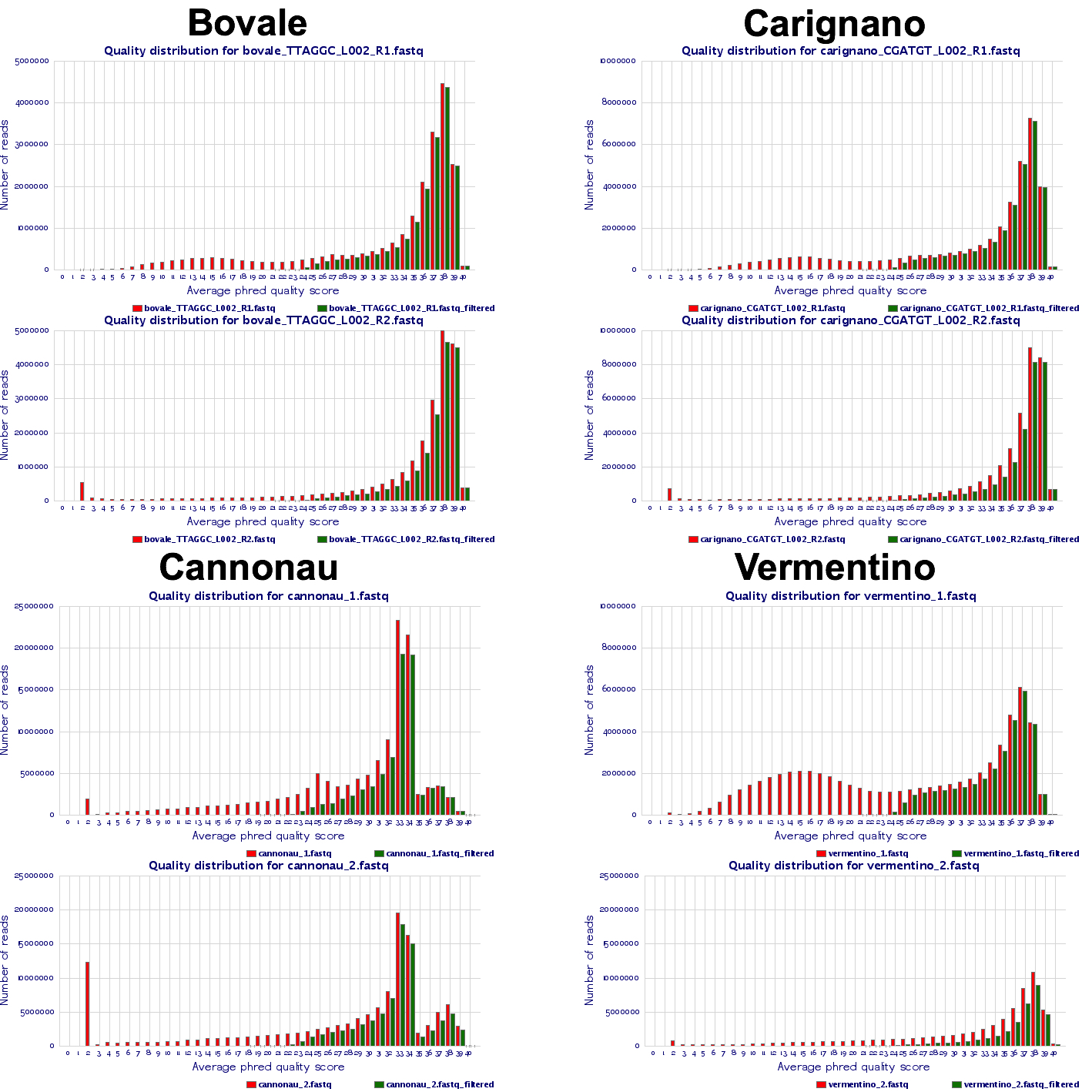


**Figure A1.** Average quality of the used reads before (red) and after (green) the quality filtering.

**Consistency of the resequencing experiment**

Resequenced cultivars were aligned to the PN40024 reference genome using bwa with an edit distance of 5%. Only position covered by at least 3 reads were selected at this stage. Mapping coverage within each chromosome was checked in order to investigate whether the produced reads were representative of the entire genome.

Figure A2 shows the average coverage for each chromosome within the 4 presented cultivars. The calculated standard deviations within the plotted data are 0.15, 1.40, 0.49 and 0.39 for cultivars Bovale, Cannonau, Carignano and Vermentino respectively.


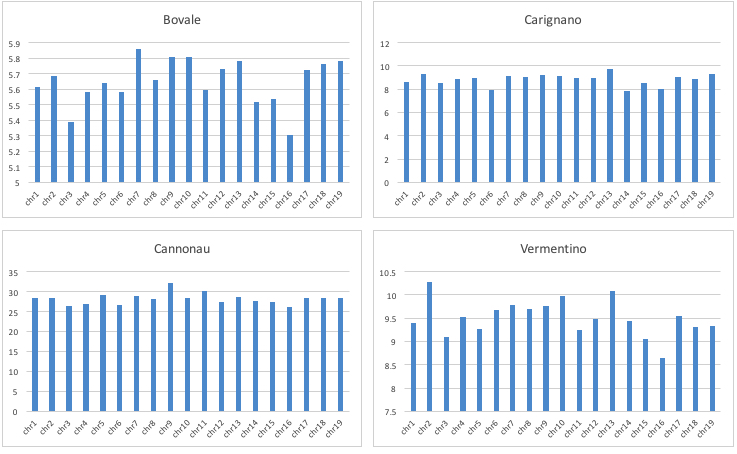


**Figure A2.** Coverage of the resequenced cultivars at chromosome level.

**Pipeline reliability by external validation**

Pipeline reliability was tested on previously published data. Polymorphisms called before the final step of quality filtering (e.g. SNPs and Indels called when the position was covered by at least 3 reads and with significance < 0.05 according to Varscan computation) proved to contain 96.3% of the detected Sultanina SNVs (Di Genova et al. 2014 BMC Plant Biology). Among these, we found 100% of the SNPs that were validated by the authors with alternate methods.

In order to test false discovery rate of the proposed method, we tested it on real Pinot cultivar PN40024 resequencing data. WGS reads for cultivar PN40024 were downloaded from the INRA website (<https://urgi.versailles.inra.fr/download/vitis/shortReads/>) and were quality filtered by using NGS QC toolkit with default parameters. Such filtered reads were aligned on the reference PN40024 reference genome by using ad edit distance of 5%. SNPs. According to the proposed method within this paper, we first called polymorphisms by applying the following settings: (a) the position was covered by at least 5 reads, (b) the polymorphism was supported by at least 3 reads, (c) the p-value calculated by Varscan was proved to be 0.05. In these condition we called as little as the 0.01% and 0.0009% of the mapped bases as SNPs and Indels respectively. These percentages dropped to 0.005 and 0.0003% respectively when the hard quality filter was applied (number of reads covering the polymorphism >= average coverage, number of reads supporting the polymorphism >= ½ * average coverage) with 27.2% of the called SNPs being homozigous. Notably, although such a result may be considered indicative of the false discovery rate of our pipeline, a better estimation may result in an even lower value possibly due to the residual heterozigosity of the highly inbred reference Pinot cultivar.
